# Public Health Response to Commercial Airline Travel of a Person with Ebola Virus Infection — United States, 2014

**Published:** 2015-01-30

**Authors:** Joanna J. Regan, Robynne Jungerman, Sonia H. Montiel, Kimberly Newsome, Tina Objio, Faith Washburn, Efrosini Roland, Emily Petersen, Evelyn Twentyman, Oluwatosin Olaiya, Mary Naughton, Francisco Alvarado-Ramy, Susan A. Lippold, Laura Tabony, Carolyn L. McCarty, Cara Bicking Kinsey, Meghan Barnes, Stephanie Black, Ihsan Azzam, Danielle Stanek, John Sweitzer, Anita Valiani, Katrin S. Kohl, Clive Brown, Nicki Pesik

**Affiliations:** 1 Division of Global Migration and Quarantine, National Center for Emerging and Zoonotic Infectious Diseases, CDC; 2 Division of Birth Defects and Developmental Disabilities, National Center on Birth Defects and Developmental Disabilities, CDC; 3 Epidemic Intelligence Service, CDC; 4 Division of Reproductive Health, National Center for Chronic Disease Prevention and Health Promotion, CDC; 5 Texas Department of State Health Services; 6 Ohio Department of Health; 7 Pennsylvania Department of Health Bureau of Epidemiology; 8 Colorado Department of Public Health and Environment; 9 Chicago Department of Public Health; 10 Nevada State Department of Health and Human Services; 11 Florida Department of Health Division of Disease Control and Health Protection; 12 Maryland Department of Health and Mental Hygiene; 13 North Carolina Department of Health and Human Services Communicable Disease Branch

Before the current Ebola epidemic in West Africa, there were few documented cases of symptomatic Ebola patients traveling by commercial airline (1,2), and no evidence of transmission to passengers or crew members during airline travel. In July 2014 two persons with confirmed Ebola virus infection who were infected early in the Nigeria outbreak traveled by commercial airline while symptomatic, involving a total of four flights (two international flights and two Nigeria domestic flights). It is not clear what symptoms either of these two passengers experienced during flight; however, one collapsed in the airport shortly after landing, and the other was documented to have fever, vomiting, and diarrhea on the day the flight arrived. Neither infected passenger transmitted Ebola to other passengers or crew on these flights (3,4). In October 2014, another airline passenger, a U.S. health care worker who had traveled domestically on two commercial flights, was confirmed to have Ebola virus infection. Given that the time of onset of symptoms was uncertain, an Ebola airline contact investigation in the United States was conducted. In total, follow-up was conducted for 268 contacts in nine states, including all 247 passengers from both flights, 12 flight crew members, eight cleaning crew members, and one federal airport worker (81 of these contacts were documented in a report published previously [5]). All contacts were accounted for by state and local jurisdictions and followed until completion of their 21-day incubation periods. No secondary cases of Ebola were identified in this investigation, confirming that transmission of Ebola during commercial air travel did not occur.

## Investigation Protocols

On October 14, 2014, the health care worker, who was among those who had cared for a patient with confirmed Ebola in the United States (6), experienced fever and rash and sought medical care. On October 15, Ebola virus infection was confirmed in this health care worker, who had traveled by commercial airline from Dallas, Texas, to Cleveland, Ohio, on October 10, 2014, and from Ohio to Texas on October 13, 2014 (Figure). The date of symptom onset was uncertain; however, based on medical history and clinical and laboratory findings, CDC determined that a contact investigation should be performed for persons aboard either flight (5).

The CDC public health response protocol for airline contact investigations involving viral hemorrhagic fevers such as Ebola involves using brief interviews about exposures and events on the flight to determine risk categories. Previously, the investigation was limited to the flight attendants and cleaning crew members who serviced the flight and to passengers seated for an extended time within 3 feet of the symptomatic passenger. This earlier protocol recommended that contacts self-monitor for fever or other symptoms for 21 days and check in weekly with the local health department, but did not recommend restrictions on travel or other activities for contacts who were asymptomatic.

Because of concern after transmission of Ebola to health care workers in Texas and recognition that data on transmission risk aboard aircraft were limited, all passengers and crew were investigated, and CDC issued additional recommendations for the investigation of the two flights between Texas and Ohio. Within 48 hours after onset of the investigation for each flight, all passengers and flight crew had been notified about the health care worker with Ebola and the ongoing investigation (Table 1). All cleaning crew members were contacted and interviewed by October 21.

## Categorization of Contacts

At the beginning of the investigation, the recommendations from CDC to state and local health departments categorized all passengers seated within 3 feet of the traveler with confirmed Ebola (the 3-foot zone) as having “some risk” (Figure). Four public health actions were recommended for these passengers. First, interview these passengers using the standard interview form. Second, initiate active, twice-daily monitoring for symptoms and fever for the 21 days following the flight; passengers were required to take their own temperature twice daily and report it to the health department once a day. Third, place these passengers in quarantine; the specific terms of quarantine were left to the discretion of the state and local jurisdictions. Fourth, place these passengers on federal public health travel restrictions (the Do Not Board list) to ensure they could not travel commercially.

Travelers seated outside the 3-foot (approximately 1 meter) zone were considered at a lower risk of exposure and were categorized in the “uncertain risk” group. Flight attendants who reported they had no known direct contact with the Ebola patient also were categorized as uncertain risk. CDC recommended that state and local health departments initiate active, twice-daily monitoring for fever and symptoms for passengers in the uncertain risk group. If people in this risk group developed symptoms, health departments were asked to complete the standard passenger or flight crew interview and contact CDC. CDC did not recommend movement or travel restrictions for passengers in the uncertain risk group, and specific guidance was at the discretion of the health departments.

If it was determined that there was no environmental contamination of the aircraft related to the Ebola patient (e.g., diarrhea or vomiting), persons who had no contact with the Ebola patient and were not within the passenger cabin (i.e., were in the cockpit) would be categorized in the “no known risk” group. This would also include the cleaning crews if no additional potential exposures were reported. The no known risk group would not require active monitoring, occupational restrictions, or travel restrictions.

In this investigation, CDC recommended that all passengers and crew members, including persons in the no known risk and uncertain risk groups, be contacted by state or local public health authorities at the end of 21 days to ensure that 1) they had remained symptom-free throughout the incubation period, or 2) any symptoms experienced were properly reported, assessed, and determined not to be caused by Ebola.

Public health actions varied by state and local jurisdiction. Many jurisdictions chose to have frequent follow-up with contacts, including those in the uncertain risk group, which in some cases included daily interaction with contacts. Other variations included requiring direct active monitoring of passengers in the 3-foot zone, which included twice-daily check-ins (once in person, and once by phone) (5,6). Although states could have issued quarantine orders for passengers in the “some risk group,” they all chose the less restrictive option of issuing guidelines to these contacts for social distancing, which typically involved avoiding congregate settings and maintaining a 3-foot distance from others.

All 268 passengers and members of the flight and cleaning crews from the two flights were contacted, interviewed, and categorized into risk groups (Table 1). Mean age of the 268 contacts was 41.4 years (range = 6 months–90 years). Of the 268 contacts, 21 (7.8%) passengers were classified as “some risk.” These included 20 passengers seated in the 3-foot contact zone during the flight and one passenger who sat within the zone for 15 minutes before exiting the aircraft (Figure). CDC placed the 20 passengers who were seated in the 3-foot contact zone during the flight on the federal Do Not Board list, and a 21-day monitoring period was initiated by their respective state public health authorities. The passenger in the some risk group because of the 15-minute exposure was not placed on the Do Not Board list; however, this person did not travel and received the same monitoring by public health authorities as others in the group. On October 27 (day 17 of monitoring for the first flight, and day 14 for the second flight), CDC’s categorization guidance was changed such that federal travel restrictions were no longer required for the passengers in the some risk group, and the 20 were removed from the Do Not Board list.

## Findings

There were no reports from the Ebola patient, flight attendants, or passengers that the patient had vomited or had diarrhea during the two flights resulting in contamination of the plane. Of the 12 persons involved in serving or cleaning the cabin, six reported wearing gloves, and one reported using hand sanitizer after picking up a few items in the cabin without wearing gloves.

Of the 268 contacts, 32 (11.9%), including 28 passengers, three flight crew members, and one member of the cleaning crew, reported within 21 days of the flight one or more symptoms that can occur with Ebola (Table 2). One passenger in the uncertain risk category experienced a fever (defined as a temperature of ≥100.4°F [≥38°C]) on day 21 of monitoring and was hospitalized the same day. The fever was accompanied by respiratory symptoms and continued for several days without a confirmed alternative diagnosis, resulting in Ebola testing on days 1 and 3 of symptoms. Both tests were negative. There were 19 passengers who had temperatures of 99.0°F (37.2°C) or higher, but <100.4°F. Of these 19 with elevated temperatures, 13 had a single episode of elevated temperature, and six had multiple episodes. Although some passengers experienced symptoms that can occur with Ebola illness during their 21-day monitoring period, the monitoring period passed with no secondary cases of Ebola found.

What is already known on this topic?

Given that transmission of Ebola occurs through direct contact with body fluids of symptomatic or deceased patients, the probability of contracting Ebola during commercial air travel is thought to be low. There have been few documented cases of Ebola patients traveling by commercial aircraft while symptomatic, and limited detail in scientific reports regarding these cases or the public health response.

What is added by this report?

A health care worker infected with Ebola virus traveled on two commercial flights within the United States before being diagnosed with Ebola. A total of 268 contacts in nine states (all 247 passengers, 12 flight crew, eight cleaning crew, and one federal airport worker) were notified and monitored for 21 days. Thirty-two persons had one or more symptoms that can occur with Ebola, but only one had symptoms that prompted Ebola testing, which was negative. No transmission of Ebola occurred on either flight.

What are the implications for public health practice?

The more inclusive approach in this investigation provided evidence that the risk for transmission of Ebola is likely low if the patient’s symptoms do not include vomiting, diarrhea, or bleeding. In cases where there is little or no environmental contamination of the aircraft, an investigation that is limited to passengers seated within 3 feet of the patient might be appropriate.

### Discussion

No secondary cases of Ebola were found in this investigation, and to date, no other airline contact investigations involving travelers with confirmed Ebola have found secondary cases among passengers or crew members (1–3,7). Guidelines for airline contact investigations for viral hemorrhagic fevers vary among countries and typically do not include notification of every passenger (7,8). When it was first learned that two U.S. health care workers using personal protective equipment had become infected with Ebola virus, CDC adopted a conservative approach for the airline contact investigation until additional information could be obtained. CDC expanded its existing airline contact investigation protocol to include all passengers, rather than limit the investigation to passengers who had been within 3 feet of the Ebola patient for a prolonged time. CDC guidance and contact investigation protocols were adapted to best protect the health of the public and address public concerns. As it became increasingly clear that Ebola transmission dynamics had not changed and transmission to passengers was not likely, the recommendations were modified to decrease restrictions on passengers within the 3-foot zone by no longer recommending that these passengers be issued quarantine orders or be added to the Do Not Board list.

Although no Ebola virus transmission occurred on these two domestic commercial flights, these findings might not be applicable to all airline contact investigations. For example, transmission during airline travel might be more likely if an exposure to body fluids from a passenger with more severe symptoms such as vomiting, diarrhea, or bleeding was to occur. In addition, both flights in this investigation were <4 hours in duration; longer flights might pose a greater risk for transmission. Previous airline contact investigations have not found evidence of Ebola transmission on commercial flights; however information about the symptoms experienced by Ebola patients aboard the aircraft in these few cases is limited (1–3,7).

This airline contact investigation provides additional evidence that the risk for Ebola transmission on commercial aircraft is likely very low when there is no evidence of blood or other body fluid exposure. Additional public health investigations and statistical modeling might be helpful to further define the possible risk for Ebola transmission on commercial flights. In future commercial flights involving Ebola-infected passengers, circumstances such as duration of exposure and degree of environmental contamination should be taken into consideration. Depending on these circumstances, limiting contact tracing to the flight crew and passengers seated within 3 feet of the Ebola patient might be appropriate.

## Figures and Tables

**FIGURE:**
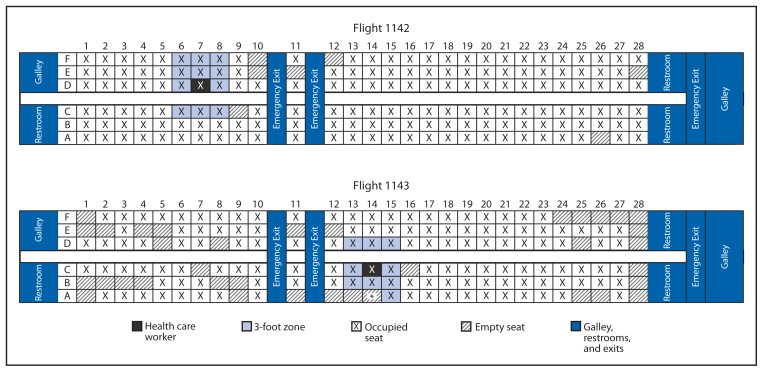
Seating charts for commercial airline flights 1142 and 1143 taken by a health care worker later diagnosed with Ebola, which became the focus of a public health response — United States, October 10 and 13, 2014 *One passenger on flight 1143 was in the 3-foot zone for only 15 minutes before exiting the plane before takeoff.

**TABLE 1:** Number of contacts (N = 268) followed from two flights taken by a health care worker later diagnosed with Ebola, by flight role — United States, October 10 and 13, 2014

Flight role	Flight 1* (Oct 10, 2014)	Flight 2† (Oct 13, 2014)	Total contacts
Passengers	164	134	**247** §
Flight crew	6	6	**12**
Cleaning crew	5	3	**8**
Airport staff	1	0	**1**
**Total contacts**	**176**	**143**	**268**

* Contacts by state of location on day 21 (Texas 122 persons, Ohio 46, Colorado 5, Illinois 1, Maryland 1, and North Carolina 1).

† Contacts by state or country of location at day 21 (Texas 93 persons, Ohio 36, Colorado 5, Ireland 2, Illinois 1, Maryland 1, Nevada 1, and North Carolina 1).

§ 51 passengers traveled on both flights.

**TABLE 2:** Symptoms reported by contacts (n = 32) from two flights within 21 days of exposure to a health care worker later diagnosed with Ebola — United States, 2014

Symptom*	Symptoms reported by 32 contacts	Symptoms reported by 21 contacts in 3-foot zone
Fever (≥100.4°F [≥38°C])	1	0
Abdominal pain	3	0
Unusual bleeding	0	0
Body aches	6	2
Diarrhea	2	0
Headache	24	3
Hiccups	0	0
Rash	1	0
Sore throat	14	2
Vomiting	0	0
Weakness	2	0

* Contacts could report more than one type of symptom.
